# Nonfebrile Seizures in Pediatrics: Key Points to Remember

**DOI:** 10.7759/cureus.53233

**Published:** 2024-01-30

**Authors:** Mariana M Anjos, Ana M Figueireido, Patricia Cardoso, Filipa Costa, Julieta Morais

**Affiliations:** 1 Pediatrics, Centro Hospitalar do Médio Tejo, Torres Novas, PRT

**Keywords:** pediatric rare diseases, rickets, alopecia, hypocalcemia, seizures

## Abstract

Seizures are the most common neurological disorder in pediatrics, and their initial approach aims at clinical stabilization. A thorough patient evaluation may provide important clues for the etiological diagnosis. A 12-month-old female child was observed in the emergency department after experiencing her first apyretic seizure. She had a history of congenital alopecia and, on physical examination, presented subtotal alopecia and milia. Initial investigation revealed hypocalcemia; therefore, intravenous calcium correction was started with a partial response. The analytical study was extended, revealing hypophosphatemia, elevated parathormone, and 1,25(OH)_2_ vitamin D with normal 25(OH) vitamin D. The genetic analysis confirmed hereditary vitamin D-resistant rickets (HVDRR). The integration of the findings was crucial to diagnostic reasoning and to guide further investigation. HVDRR is a rare disorder, with more severe clinical presentations associated with alopecia. Early diagnosis and treatment are fundamental to minimize the impact on growth and the development of other comorbidities.

## Introduction

Seizures are the most common neurological disorder in pediatrics [1] and can be classified as unprovoked or acute symptomatic [2]. The initial approach to the patient aims at clinical stabilization followed by thorough anamnesis and a physical examination that can provide important clues to determine the etiological diagnosis [1,3]. An acute symptomatic seizure results from a CNS insult [3,4]. To define such an association, it is important to investigate, either by clinical or laboratory findings, if there are any signs of CNS trauma, infection, vascular issues, toxic exposure, or metabolic dysfunction [1,3-5]. Electrolyte disturbances represent a frequent cause of nonfebrile seizures at any age, and the most common electrolyte imbalances implicated are hyponatremia, hypocalcemia, and hypomagnesemia [4].

## Case presentation

A 12-month-old female child was brought to the emergency department after an episode of sudden hypotonia, loss of consciousness, perioral cyanosis, sialorrhea, an empty gaze, and tremors of the limbs. The episode was brief and was followed by subsequent drowsiness. There was no previous context of illness.

The patient had a history of congenital alopecia and acute pyelonephritis at four months old. She had adequate psychomotor development and registered normal weight and height growth, except for the head circumference, which was above the 97th percentile since she was six months old. She took a vitamin D supplement inconsistently in the first year of life.

**Figure 1:**
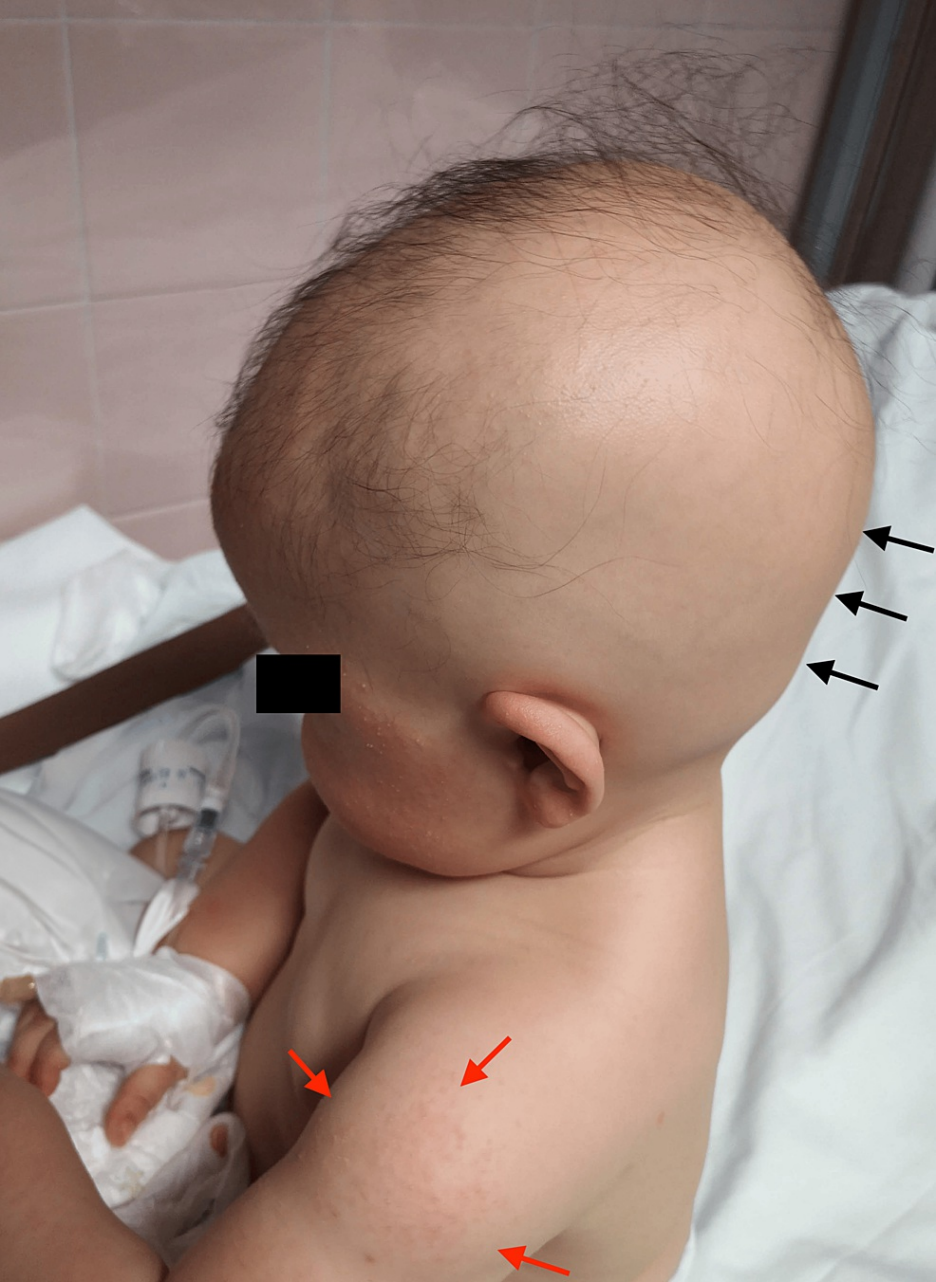
Subtotal alopecia and milia on the arms Black arrows: subtotal alopecia; red arrows: milia on the arms

On physical examination, beyond macrocrania and an enlarged anterior fontanelle, which was about 2 cm, she had a normal neurological exam. Subtotal alopecia and milia on the face and arms were noticed (Figure 1). 

Initial blood tests revealed a normal RBC and WBC count and CRP, hypocalcemia (ionized calcium 0.76 mmol/L, reference range (RR): 1-1.3 mmol/L), increased alkaline phosphatase (1,075 IU/L, RR: 311-884 IU/L), and increased lactate dehydrogenase (520 IU/L, RR: 150-300 UI/L). The ECG showed no abnormalities.

Correction of hypocalcemia was initiated with an intravenous infusion of 10% calcium gluconate, and despite multiple doses, normocalcemia could not be achieved.

Further etiological investigation revealed hypophosphatemia (1.25 mmol/L, RR: 1.4-2.1 mmol/L), elevated parathormone (374.6 pg/mL, RR: 8-51 pg/mL), 1,25(OH)_2 _vitamin D (250 pmol/L, RR: 39-193 pmol/L), and bone alkaline phosphatase (369 ug/L, RR: 28-187 ug/L) with normal 25(OH) vitamin D levels. The complete laboratory workup is shown in Table 1.

**Table 1:** Laboratory results for a 12-month-old girl with a new-onset seizure H: high; L: low; N: normal

Laboratory test	Value	Interpretation	Reference range
Complete blood cell count			
Hemoglobin	12.3 g/dL	N	10.6-14.5 g/dL
WBC	13,130/uL	N	6,000-16,000/uL
Neutrophils	6,540/uL	N	6,000-5,100/uL
Lymphocytes	5,160/uL	N	2,700-12,000/uL
Platelets	420,000/uL	N	150,000-450,000/uL
Serum chemistry			
Sodium	136 mmol/L	N	136-146 mmol/L
Potassium	5.1 mmol/L	N	3.5-5.1 mmol/L
Chloride	102 mmol/L	N	101-109 mmol/L
﻿Bicarbonate	22.4 mmol/L	N	21-28 mmol/L
﻿Blood urea nitrogen	16 mg/dL	N	17-43 mg/dL
﻿Creatinine	0.4 mg/dL	N	0.4-0.7 mg/dL
﻿Glucose	101 mg/dL	N	74-106 mg/dL
﻿Magnesium	2.1 mg/dL	N	1.9-2.5 mg/dL
﻿Phosphorus	1.25 mmol/L	L	1.4-2.1 mmol/L
﻿Calcium	1.66 mmol/L	L	2.3-2.65 mmol/L
Ionized calcium	0.76 mmol/L	L	1-1.3 mmol/L
﻿Albumin	5.0 g/dL	N	3.5-5.2 g/dL
﻿Lactate	2.3 mmol/L	H	0-1.3 mmol/L
CRP	1.33 mg/dL	N	<2 mg/dL
Lactate dehydrogenase	520 IU/L	H	150-300 UI/L
Alkaline phosphatase	1,075 IU/L	H	311-884 IU/L
Bone alkaline phosphate	369 ug/L	H	28-187 ug/L
Parathormone	374.6 pg/mL	H	8-51 pg/mL
1,25(OH)_2_ vitamin D	250 pmol/L	H	39-193 pmol/L
25(OH) vitamin D	16.4 ng/mL	N	Deficiency <10 ng/mL
﻿Thyroid-stimulating hormone	2.502 uUI/mL	N	2.3 +/- 1.6 uUI/mL
T4	0.8 ng/dL	N	0.6-1.1 ng/dL

**Figure 2:**
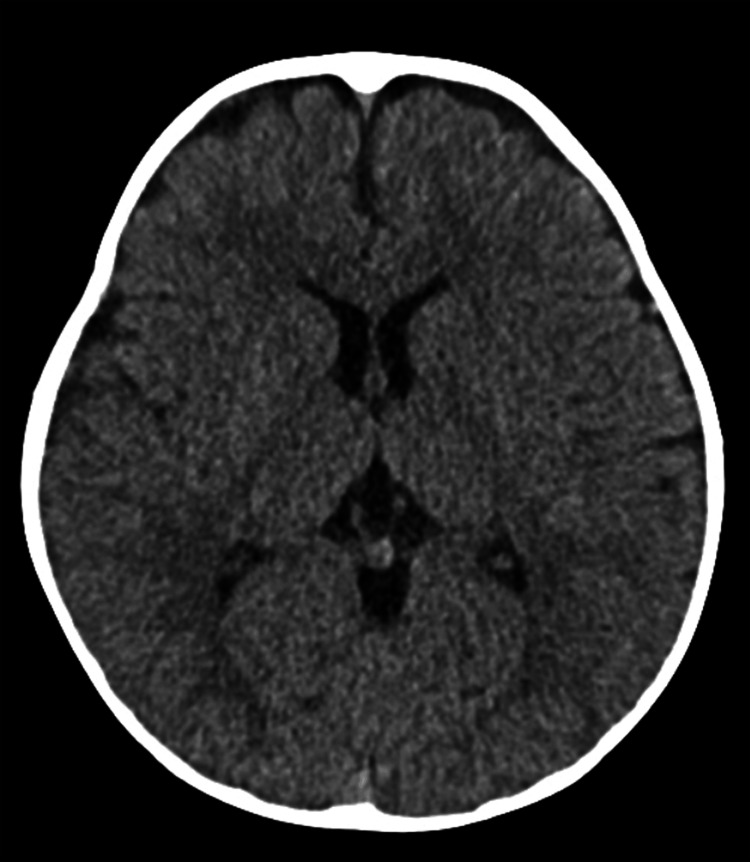
Normal head tomography

**Figure 3:**
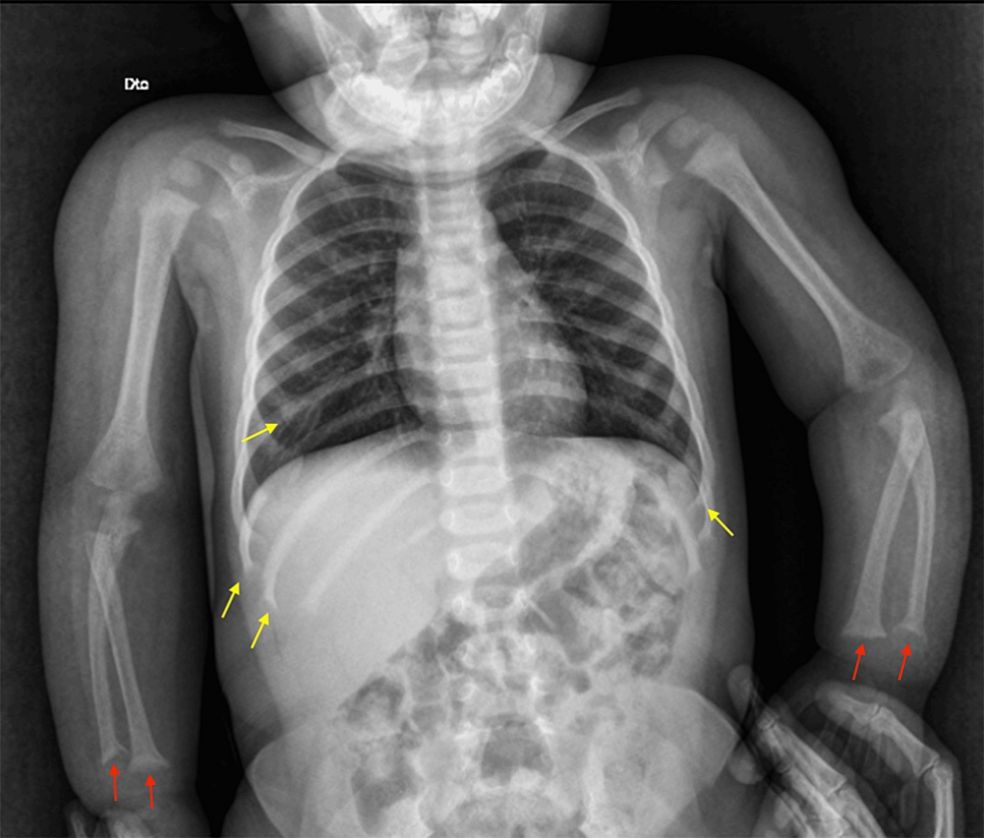
Skeletal survey of the upper limbs and thorax Red arrows: bilateral flattening and irregularities of the distal epiphyses of the radius and ulna; yellow arrows: rachitic rosary, visible on the extremities of the ribs

**Figure 4:**
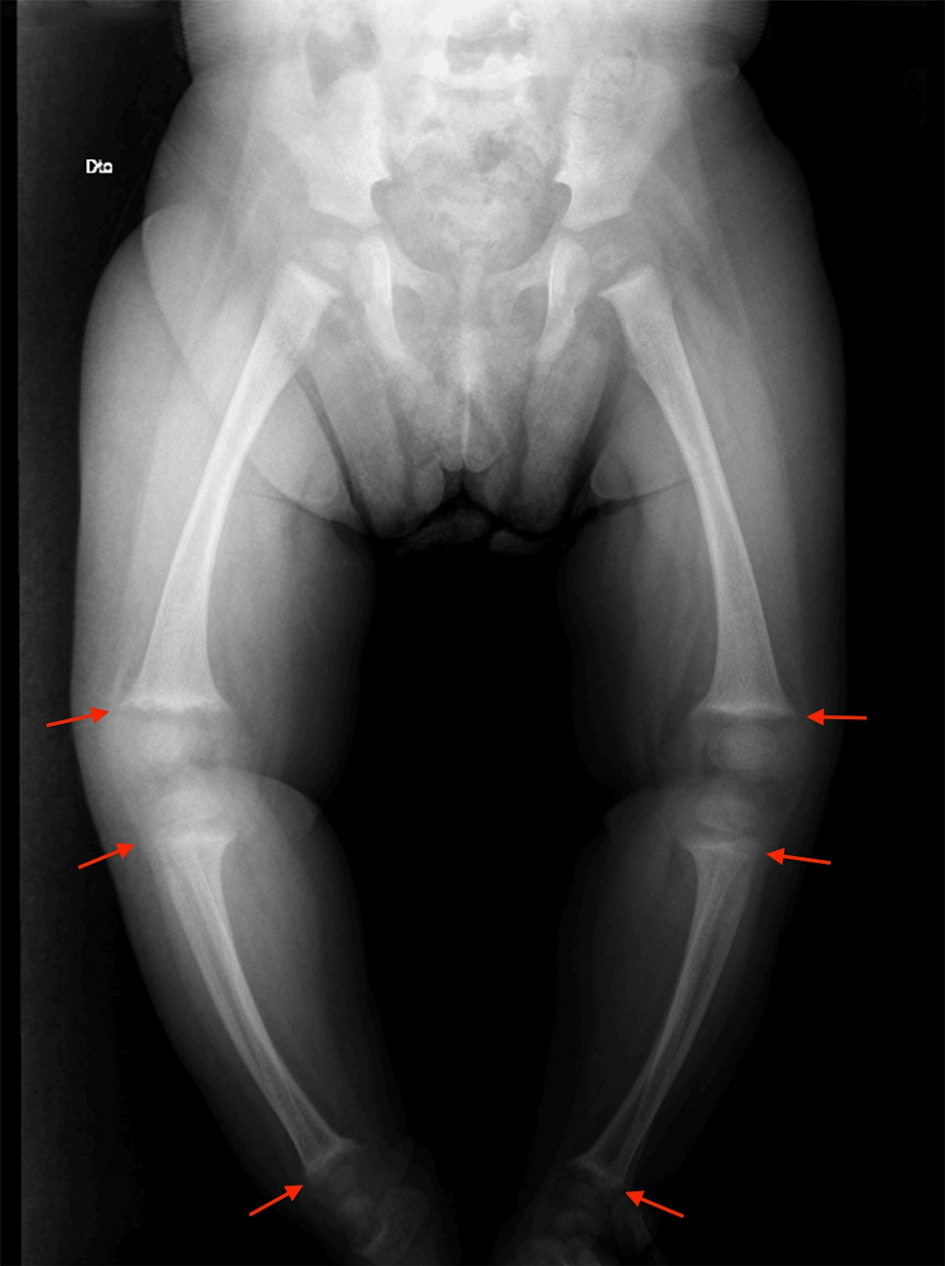
Skeletal survey of the lower limbs Red arrows: bilateral flattening and irregularities of the proximal epiphyses of the tibia and the distal epiphyses of the femur and tibia

The clinical picture was suspicious for hereditary vitamin D-resistant rickets (HVDRR) type IIA, and the patient was transferred to a tertiary center for further management. Further investigation revealed a normal EEG and head tomography (Figure 2), and a skeletal survey showed findings compatible with rickets (Figures 3, 4).

A genetic study confirmed the diagnosis of HVDRR type IIA, showing a homozygous variant on the vitamin D receptor (VDR; c.133A>G p.(Lys45Glu)).

## Discussion

Acute symptomatic seizures can be caused by various conditions that result in CNS insults, with metabolic and electrolyte abnormalities being some of the more frequent and treatable causes of pediatric seizures [2,3,5].

In the initial approach to a seizure, the priority is to stabilize the patient and look for reversible causes. So, beyond the search for signs of other etiologies that require specific treatment, it is also advisable to look for electrolyte and metabolic imbalances and promptly correct them [4].

In the case described, the initial workup revealed hypocalcemia. There were no clinical or lab abnormalities to point to a toxic, infectious, or inflammatory cause of the seizure. The structural cause of the seizure was remote since there were no clinical signs of intracranial hypertension or focal neurological deficits [5].

Hypocalcemia can be a result of various conditions, such as hypoparathyroidism, low vitamin D levels, medication or toxic substances, alkalosis or hypomagnesemia, or severe illness [5]. The child did not take any medication; other ion concentrations were normal, and she did not appear to be intoxicated. The elevated alkaline phosphatase suggested vitamin D deficiency as the cause of the hypocalcemia [6]. The integration of the patient history, physical examination, and initial lab abnormalities was crucial to the diagnostic reasoning and to guide further management.

Rickets is a disease caused by calcium, phosphate, or vitamin D deficiency, leading to inadequate mineralization of osteoid tissue in the growth plate and bone matrix [7]. Patients with rickets have similar clinical manifestations, such as irritability, fatigue, muscle cramps, seizures, craniotabes, delayed closure of fontanelles, frontal bossing, enlarged wrists, bowed legs, short stature, and bone pain [7]. Our patient presented with a first apyretic seizure, hypocalcemia, and macrocrania with a wide anterior fontanelle.

The most frequent cause of rickets in children globally is nutritional vitamin D deficiency [7]. In developed countries, most infants that present hypocalcemic seizures are more likely to have an underlying endocrinological etiology than dietary insufficiencies [4].

Genetic causes of rickets are rare [7]. HVDRR type IIA is a rare autosomal recessive disorder with resistance to 1,25(OH)_2_D as a result of mutations in the VDR (located in 12q13.11) [7,8]. The defect in the VDR gene causes abnormal calcium absorption in the intestine [8]. Sparse body hair or total alopecia, multiple milia, and dermoid cysts are found in the majority of patients with severe clinical presentations and resistance to vitamin D treatment associated with alopecia [8-11].

Further investigation regarding the cause of rickets revealed elevated parathormone, 1,25(OH)_2_ vitamin D, and bone alkaline phosphate with normal 25(OH) vitamin D. These results, as well as the clinical manifestations (alopecia and milia), supported the diagnosis of HVDRR type IIA, which was confirmed by genetic testing.

Initial management of hypocalcemia requires prompt intravenous correction [9]. Severe cases of HVDRR may need high doses of intravenous calcium, which can result in long-term complications like cardiac arrhythmia, hypercalciuria, nephrocalcinosis, catheter-related sepsis, and extravasation [12]. In mild to moderate cases, treatment consists of high doses of oral calcitriol along with supplemental calcium, as patients are usually more resistant to standard therapy [11-13].

## Conclusions

The approach to seizure management should target patient stabilization and the determination of any acute symptomatic etiologies. Electrolyte disturbances, as a frequent and treatable cause of pediatric seizures, must be ruled out. An accurate evaluation of the child and its personal history may provide diagnostic clues, and the integration of physical exam findings should guide further investigation. HVDRR is a rare disorder that manifests with hypocalcemia, with more severe clinical presentations associated with alopecia. Early diagnosis and treatment are fundamental to minimize the impact on growth and the development of additional comorbidities.
